# Supplementary material for: Dermatan Sulfate Is a Potential Regulator of IgH via Interactions With Pre-BCR, GTF2I, and BiP ER Complex in Pre-B Lymphoblasts
Source: Front Immunol. 2021 May 25;12:680212. doi: 10.3389/fimmu.2021.680212 (PMC8185350; doi:10.3389/fimmu.2021.680212)
Supplement: Supplementary file 1 [file DataSheet_1.pdf]

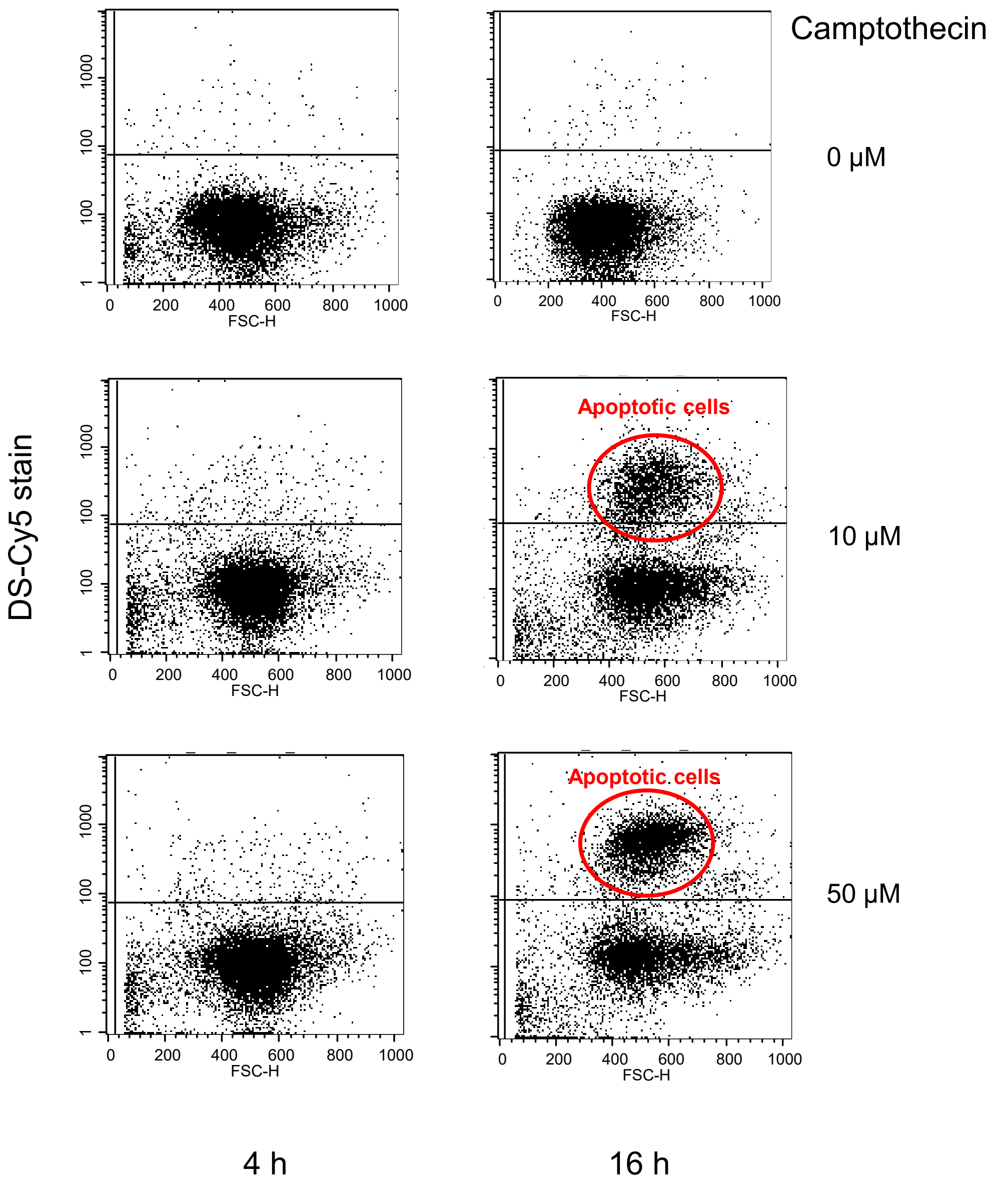

Suppl. Fig. 1. NFS-25 cells cultured with camptothecin and stained with DS-Cy5 to demonstrate the binding of DS to apoptotic cells (red circles).
