# Supplementary material for: Dermatan Sulfate Is a Potential Regulator of IgH via Interactions With Pre-BCR, GTF2I, and BiP ER Complex in Pre-B Lymphoblasts
Source: Front Immunol. 2021 May 25;12:680212. doi: 10.3389/fimmu.2021.680212 (PMC8185350; doi:10.3389/fimmu.2021.680212)
Supplement: Supplementary file 2 [file DataSheet_2.pdf]

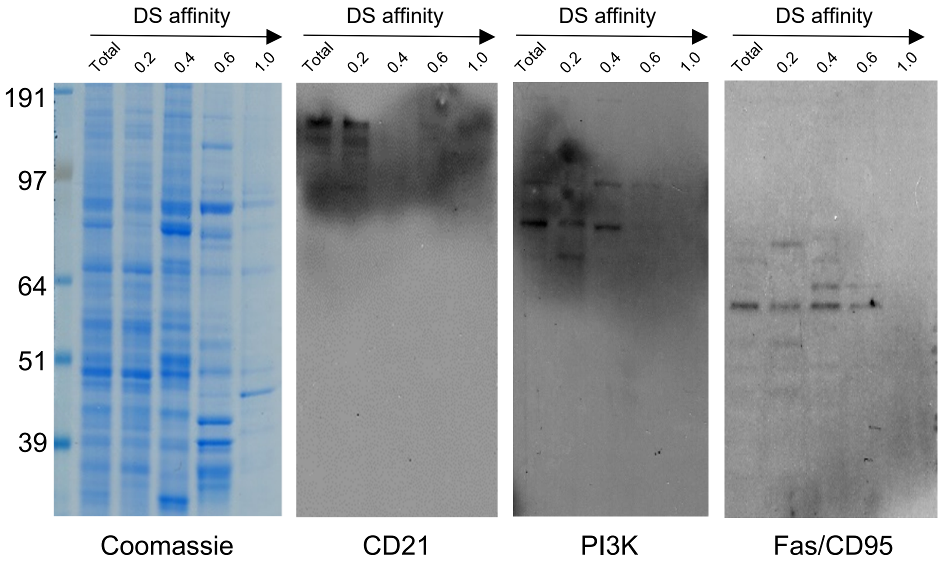


**Suppl. Fig. 2.** NFS-25 cell proteins were fractionated by increasing DS affinity (left to right) and blotted with anti-CD21, anti-PI3K, and anti-Fas (CD95).
