# Supplementary material for: Dermatan Sulfate Is a Potential Regulator of IgH via Interactions With Pre-BCR, GTF2I, and BiP ER Complex in Pre-B Lymphoblasts
Source: Front Immunol. 2021 May 25;12:680212. doi: 10.3389/fimmu.2021.680212 (PMC8185350; doi:10.3389/fimmu.2021.680212)
Supplement: Supplementary file 4 [file DataSheet_4.pdf]

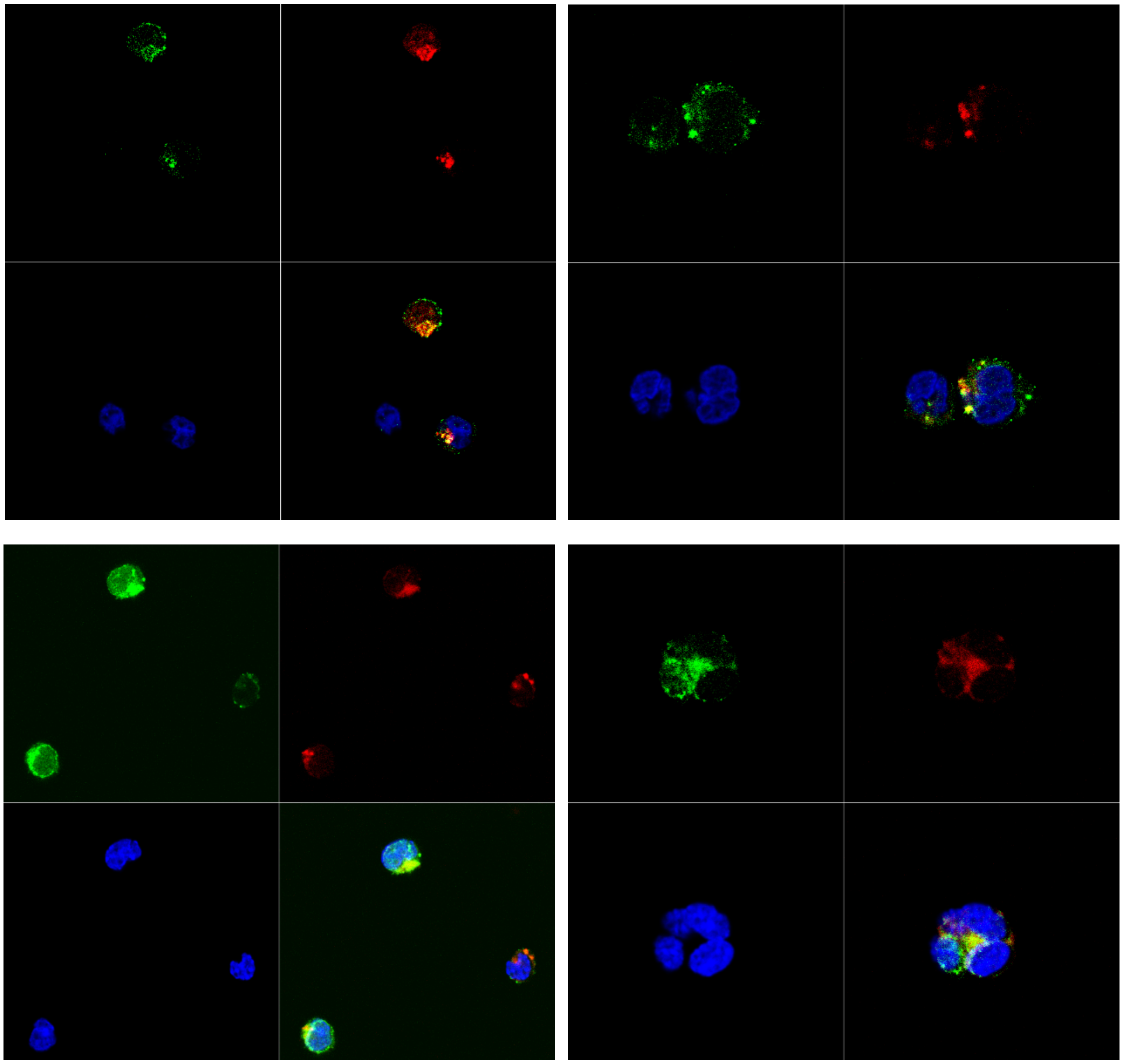

Suppl. Fig. 4. NFS-25 cells cultured with DS-AF568 (red) and stained with anti-IgH μ (green) and DAPI. Each panel shows four quadrants: green (upper left), red (upper right), blue (lower left), and merged (lower right) channels.
