# Supplementary material for: Dermatan Sulfate Is a Potential Regulator of IgH via Interactions With Pre-BCR, GTF2I, and BiP ER Complex in Pre-B Lymphoblasts
Source: Front Immunol. 2021 May 25;12:680212. doi: 10.3389/fimmu.2021.680212 (PMC8185350; doi:10.3389/fimmu.2021.680212)
Supplement: Supplementary file 5 [file DataSheet_5.pdf]

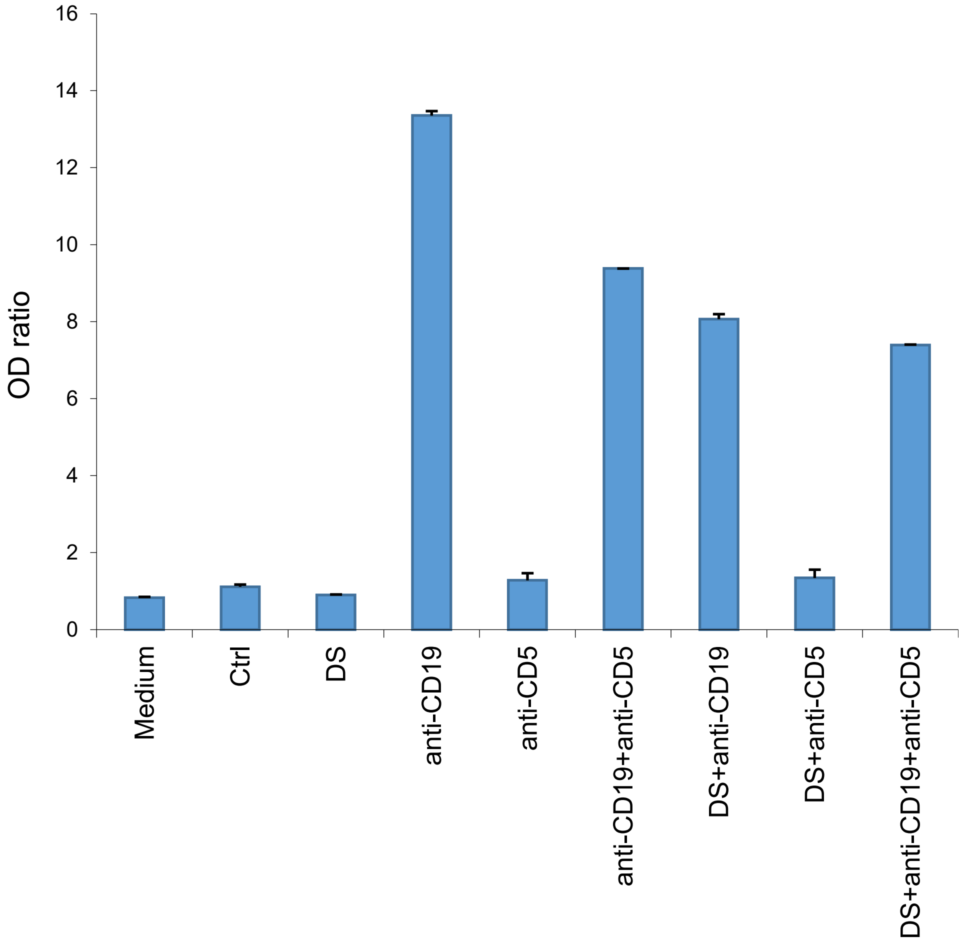

Suppl. Fig. 5. Mesurement of IgH μ secretion by NFS-25 cells cultured with various stimulants. Y axis numbers are the ratios of average $OD_{405\ nm}$ readings of wells coated with anti-IgH μ vs. wells without anti-IgH μ coating (blank controls). Medium: culture medium without cells. Ctrl: control cells cultured with medium only.
