# Supplementary material for: Dermatan Sulfate Is a Potential Regulator of IgH via Interactions With Pre-BCR, GTF2I, and BiP ER Complex in Pre-B Lymphoblasts
Source: Front Immunol. 2021 May 25;12:680212. doi: 10.3389/fimmu.2021.680212 (PMC8185350; doi:10.3389/fimmu.2021.680212)
Supplement: Supplementary file 6 [file DataSheet_6.pdf]

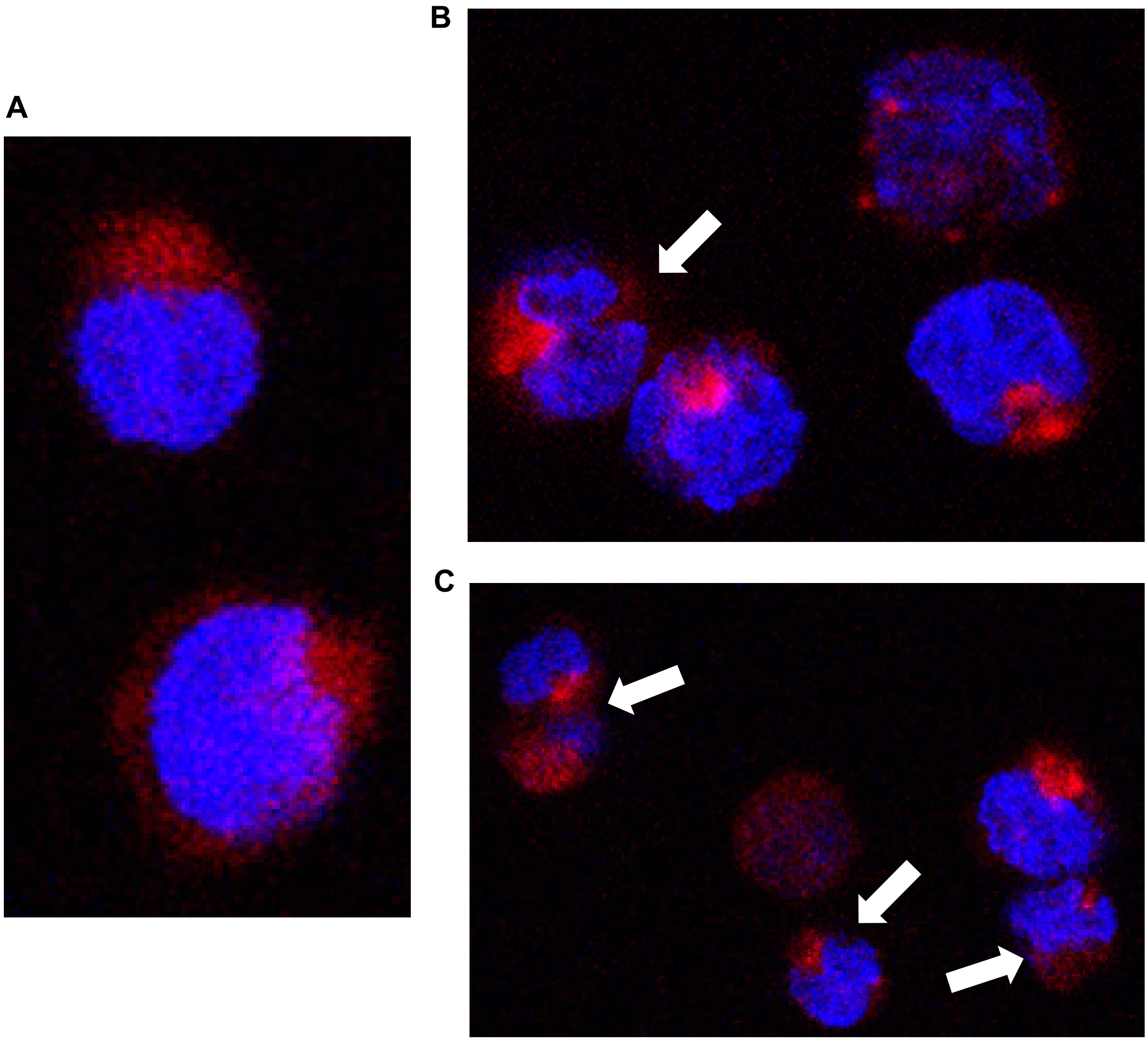

Suppl. Fig. 6. DS-interacting ER stress proteins appear to be involved in both viable cells (**A**) and apoptotic/dying cells (**B**-**C**, white arrows). NFS-25 cells cultured with DS-AF568 (**A**) and with DS-AF568 and CPT apoptosis inducer (**B** and **C**). Red: DS-AF568; Blue: nuclei stained with DAPI. White arrows indicate dying or dead cells.
