# Supplementary material for: Dermatan Sulfate Is a Potential Regulator of IgH via Interactions With Pre-BCR, GTF2I, and BiP ER Complex in Pre-B Lymphoblasts
Source: Front Immunol. 2021 May 25;12:680212. doi: 10.3389/fimmu.2021.680212 (PMC8185350; doi:10.3389/fimmu.2021.680212)
Supplement: Supplementary file 7 [file DataSheet_7.pdf]

Supplemental Table 1. Proteins identified from NSF-25 cells with strong DS affinity.

Number of peptides	Gene	Protein and compartment
		**Endoplasmic Reticulum (ER)**
37	Hspa5	BiP (Ig heavy chain-binding protein), GRP-78
54	Hsp90b1	Endoplasmin, polymorphic tumor rejection antigen 1, tumor rejection antigen gp96, Grp94, Tra-1
31	Hsp90ab1	Heat shock protein HSP 90-beta; Hsp84; Hsp84-1, Hspcb
21	Hspd1	60 kDa heat shock protein (Hsp60), chaperonin 60 (CPN60), mitochondrial matrix protein 1
16	Hspa8	Heat shock cognate 71 kDa protein, Hsc70, Hsc73
8	Hspa9	Stress-80 protein, Grp75, Hsp74, Hspa9a
35	Ganab	Neutral alpha-glucosidase AB, alpha-glucosidase 2, G2an
14	Prkcsh	Glucosidase 2 subunit beta, protein kinase C substrate 60.1 kDa protein heavy chain
22	Vcp	Transitional endoplasmic reticulum ATPase, valosin-containing protein
18	Canx	Calnexin
17	Kpnb1	Importin subunit beta-1, pore targeting complex 97 kDa subunit, Impnb
16	Pdia3	Protein disulfide-isomerase A3, ER resident protein 57 or 60 (ERp57, ERp60), GRP58
15	P4hb	Protein disulfide-isomerase, ER resident protein 59 (Erp59), Pdia1, cellular thyroid hormone-binding protein
14	Pdia4	Protein disulfide-isomerase A4
8	Rpn2	Dolichyl-diphosphooligosaccharide-protein glycosyltransferase subunit 2, Ribophorin-2, HAP6
7	Cct8	T-complex protein 1 subunit theta, Cctq
6	Cct3	T-complex protein 1 subunit gamma, Matricin, CCT-gamma, Cctg
		**Nucleus**
24	Ncl	Nucleolin
11	Hsp90aa1	Heat shock protein HSP 90-alpha, tumor-specific transplantation 86 kDa antigen, Hsp86, Hspca
12	Xrcc6	X-ray repair cross-complementing protein 6, Ku70 autoantigen, ATP-dependent DNA helicase 2
9	Xrcc5	X-ray repair cross-complementing protein 5, Lupus Ku86 autoantigen, ATP-dependent DNA helicase 2 subunit 2, CTC85
12	Prmt5	Protein arginine N-methyltransferase 5, histone-arginine N-methyltransferase, Jbp1, Skb1
11	Lmnb1	Lamin-B1
10	Eftud2	116 kDa U5 small nuclear ribonucleoprotein component, Snrp116
10	Supt16h	FACT complex subunit SPT16, Fact140
8	Ssrp1	FACT complex subunit, structure specific recognition protein 1, recombination signal sequence recognition protein 1
7	Supt5h	Transcription elongation factor SPT5; DRB sensitivity-inducing factor large subunit
10	Mfap1b	Microfibrillar-associated protein 1B, splicesome B complex protein MFAP1B
8	Pkm	Pyruvate kinase PKM, Pkm2, Pk3, Pykm
8	Hnrnpu	Heterogeneous nuclear ribonucleoprotein U, scaffold-attachment factor A
7	Nasp	Nuclear autoantigenic sperm protein
7	Mcm6	DNA replication licensing factor MCM6
6	Actn4	Alpha-actinin-4
6	Nap1l1	Nucleosome assembly protein 1-like 1, brain protein DN38, Nrp
6	Pabpc1	Polyadenylate-binding protein 1
		**Other**
13	Eif3b	Eukaryotic translation initiation factor 3 subunit B, eIF-3-eta, Eif3s9
6	Snx2	Sorting nexin-2
10	Eif3l	Eukaryotic translation initiation factor 3 subunit L
10	Tfrc	Transferrin receptor 1, CD71, TfR1
7	Lck	Proto-oncogene tyrosine-protein kinase LCK, leukocyte C-terminal Src kinase (LSK), lymphocyte cell-specific protein-tyrosine kinase, Lsk-t
